# Nasal Dermoid Cyst: A Case Report

**DOI:** 10.7759/cureus.21725

**Published:** 2022-01-30

**Authors:** Khalid Al Hawsawi, Ashwaq K Alosaimi, Atheer Aljohani, Lein Azzhary, Norah Algethami

**Affiliations:** 1 Dermatology, King Abdulaziz Hospital, Makkah, SAU; 2 Dermatology, Umm Al-Qura University, Makkah, SAU; 3 Medicine, Umm Al-Qura University, Makkah, SAU

**Keywords:** dermoid cyst, nasal dermoid cyst, nasal glioma, skin lesion, encephalocele

## Abstract

A dermoid cyst (DC) is a rare, benign congenital skin lesion that can occur anywhere on the skin and take any shape. DC is clinically diagnosed through imaging and biopsy. The usual treatment of choice for DC is surgical excision to prevent any local complications, such as inflammation, infection, and bone resorption. The type of surgery depends on the size, location, and extension of the cyst. When the cyst presents in certain areas, such as the nose, face, and scalp, surgery can be difficult owing to the possibility of an intracranial connection. Therefore, imaging is usually performed before intervening surgically. Here, we present the case of a two-year-old boy with no medical or surgical history who presented to our dermatology clinic with a slow-growing mass on his nose. During the consultation, the mass was examined, a complete medical history was obtained, and the patient was advised to undergo imaging, which revealed that the mass was a DC. Nevertheless, no deep connection was observed on imaging, and the mass was surgically removed without any complications.

## Introduction

A dermoid cyst (DC) is a benign, cystic lesion formed when the ectoderm fails to separate from the neural tube. It is lined by stratified squamous epithelium and typically presents on the neck, scalp, and face [1]. DC usually presents as a painless, slow-growing lesion that is diagnosed within the age of 1-3 years [1,2].

During the surgical removal of DC, intracranial communication is possible; therefore, an exclusion is required before biopsy or surgical intervention [2]. When a patient presents with a mass near to or at the facial midline, it is critical to consider other differential diagnoses that might be similar in clinical presentation, such as nasal glioma, lipoma, and encephalocele [3]. The use of magnetic resonance imaging (MRI) helps not only in the diagnosis of DC but also in ruling out the presence of any intracranial communication [3,4]. Here, we report the case of a congenital nasal DC in a concerning location near the midline.

## Case presentation

A two-year-old boy was brought to our clinic by his parents who were concerned about the presence of a growing mass on the upper right side of the child’s nose since birth. A detailed history of the patient revealed that he lived a healthy life devoid of any medical complications or surgery. However, the mass continued to slowly increase in size with age. Prenatal, natal, and postnatal histories were unremarkable. Family history did not reveal any similar conditions, and the parents were not consanguineous.

**Figure 1:**
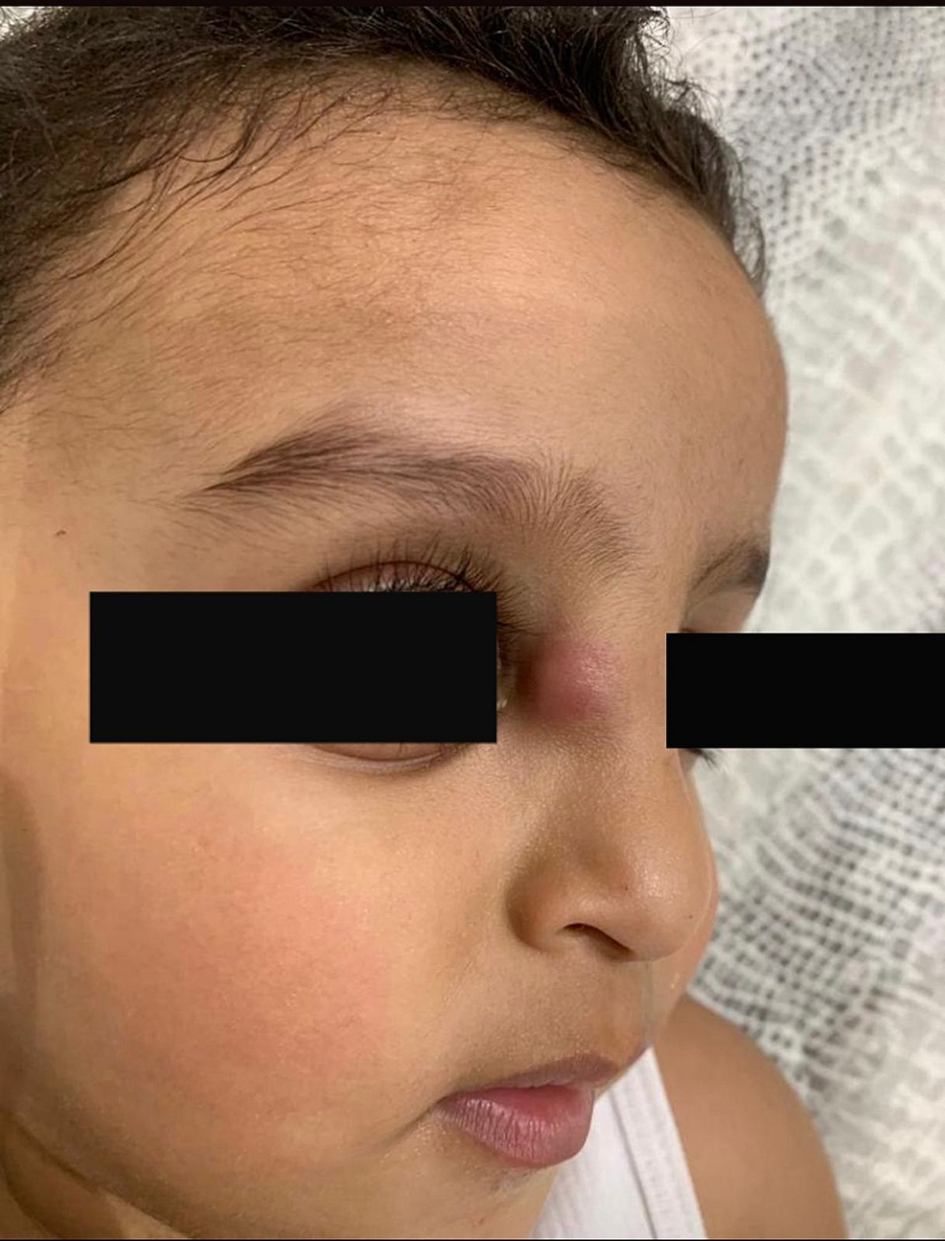
The dace of the child showing 1 × 1 cm dome-shaped, erythematous, nontender, firm, noncompressible, subcutaneous nodule on the upper right side of the nasal bridge.

**Figure 2:**
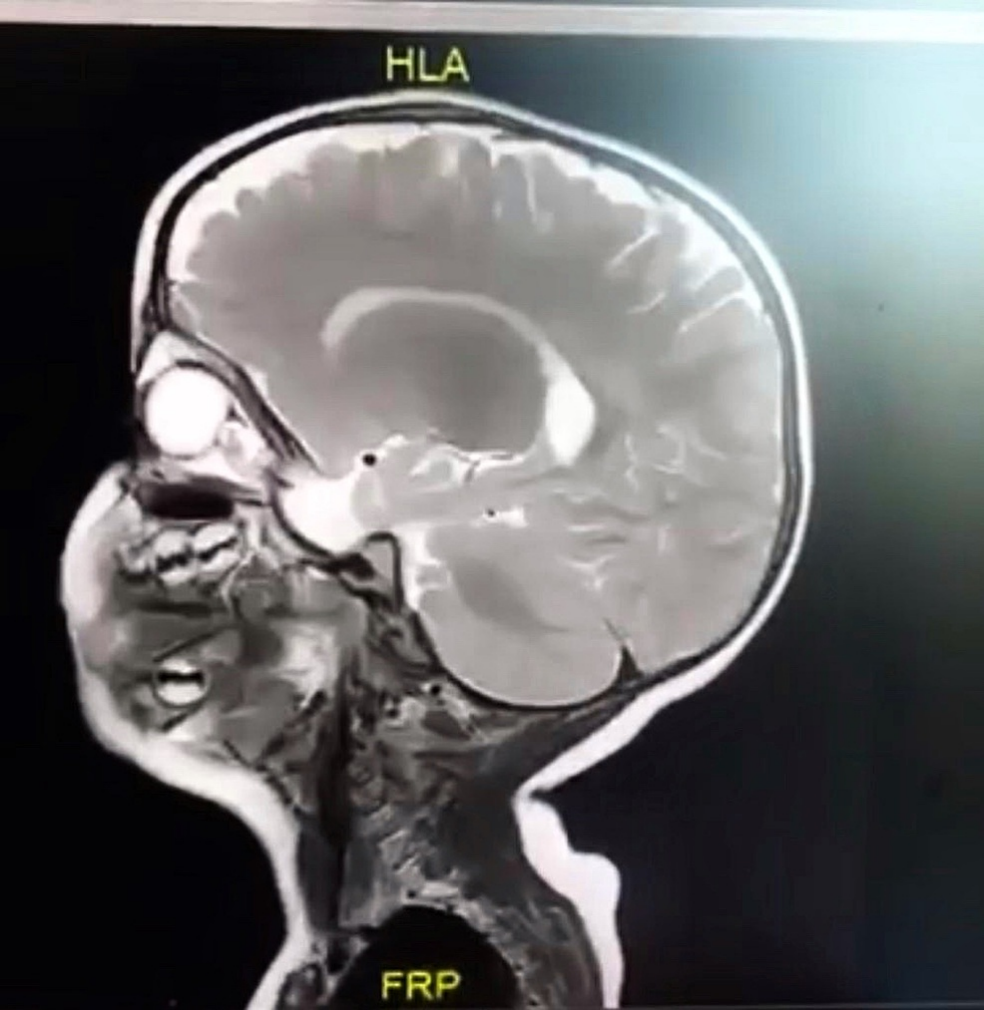
MRI of the face showing the nasal dorsum lesion measuring 10 × 16 × 10 mm with draining sinus tract anterior to the nasofrontal junction with no intracranial extension. MRI: magnetic resonance imaging

Skin examination showed a 1 × 1 cm dome-shaped, subcutaneous nodule on the upper right side of the nasal bridge that was erythematous, firm, noncompressible, and nontender (Figure 1). The transillumination test was negative. No other skin lesions were found elsewhere on the body. The examination of the hair, nails, and mucous membranes was normal. Differential diagnoses included nasal glioma, encephalocele, congenital hemangioma, sebaceous cyst, and lipoma. MRI of the facial region with contrast showed a nasal dorsum lesion measuring 10 × 16 × 10 mm with a draining sinus tract anterior to the nasofrontal junction with no intracranial extension (Figure 2).

**Figure 3:**
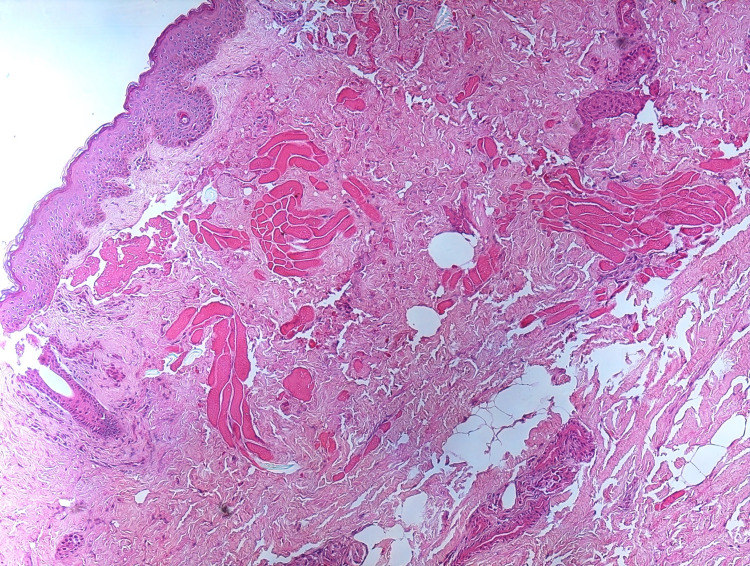
Histopathologic features of the excised skin lesion showing a polypoidal tissue covered by unremarkable keratinized stratified squamous epithelium with underlying skin adnexal including hair follicles and sebaceous glands (ectodermal in origin). Lobules of mature adipose tissue and skeletal muscle bundles separated by a thin, fibrous capsule (mesodermal in origin) were also noted.

Based on these findings and clinical presentation, a diagnosis of DC without intracranial extension was made. The patient was referred to a pediatric surgeon, and the lesion was excised and sent for histopathological examination. The histopathology report confirmed the diagnosis of DC, showing a polypoidal tissue covered by unremarkable keratinized stratified squamous epithelium and underlying skin adnexal, including hair follicles and sebaceous glands (ectodermal in origin). Lobules of mature adipose tissue and skeletal muscle bundles separated by a thin, fibrous capsule (mesodermal in origin) were also noted (Figure 3).

## Discussion

A nasal DC is a rare congenital and developmental condition. It presents as a nontender, mobile subcutaneous nodule. Although it commonly appears around the ages of 1-3 years, it can appear at any age [1,5]. It can also occur anywhere on the skin, deep under the skin, or in the interosseous space [5]. The most common location is in the orbital ridge region, with no association or deep extension [3,6]. Approximately 3% of DCs are located in the nasal midline, including the glabella, nasal dorsum, and columella. Because of the connection between the nasal and cranial cavities, the presence of deep extension and central nervous system communication is possible. Therefore, MRI should be performed for midline masses before further invasive procedures to prevent complications, such as meningitis [5,7]. Moreover, some experts recommend performing imaging for lesions close to the midline area [3,5]. In our case, the DC was present near the nasal midline; however, imaging did not show any intracranial extension or communication. Therefore, surgical excision could be safely performed by a pediatric surgeon. In addition, the removed cyst was histopathologically analyzed, which confirmed the diagnosis of DC.

The differential diagnoses in our patient included nasal glioma, encephalocele, congenital hemangioma, sebaceous cyst, and lipoma [5,8]. A nasal glioma is an ectopic, neuroectoderm from early development that presents as a firm, noncompressible, flesh-colored nodule. It is typically found at the root of the nose [6,9]. In contrast, an encephalocele is a herniation of cranial contents through a defect in the skull. It is formed when the separation of the neuroectoderm from the surface ectoderm fails during embryogenesis [6,10]. Encephalocele is usually found at the occiput, dorsal nose, orbit, or forehead. It is characterized by an increase in size when the child cries, compressibility to palpation, and a positive transillumination test [5,6,11].

## Conclusions

Nasal DC is a rare congenital skin lesion that should be differentiated from nasal glioma and encephalocele. Imaging studies should be performed for midline masses to rule out intracranial connections and prevent the occurrence of complications. In our case, the presenting DC was near the midline; therefore, imaging was performed with caution before any surgical intervention.
